# Varian HDR surface applicators — commissioning and clinical implementation

**DOI:** 10.1120/jacmp.v17i2.6033

**Published:** 2016-03-08

**Authors:** Ileana Iftimia, Andrea B. McKee, Per H. Halvorsen

**Affiliations:** ^1^ Radiation Oncology Department Lahey Hospital and Medical Center Burlington MA; ^2^ Tufts University School of Medicine Boston MA USA

**Keywords:** surface applicators, source vertically positioned

## Abstract

The purpose of this study was to validate the dosimetric performance of Varian surface applicators with the source vertically positioned and develop procedures for clinical implementation. The Varian surface applicators with the source vertically positioned provide a wide range of apertures making them clinically advantageous, though the steep dose gradient in the region of 3‐4 mm prescription depth presents multiple challenges. The following commissioning tests were performed: 1) verification of functional integrity and physical dimensions; and 2) dosimetric measurements to validate data provided by Varian as well as data obtained using the Acuros algorithm for heterogeneity corrected dose calculation. A solid water (SW) phantom was scanned and the Acuros algorithm was used to compute the dose at 5 mm depth and at surface for all applicators. Two sets of reference dose measurements were performed, with the source positioned at (i) −10 mm and (ii) −15 mm from the center of the first nominal dwell position. Measurements were taken at 5 mm depth in a SW phantom and in air at the applicator surface. The results were then compared to the vendor's data and to the Acuros calculated dose. Relative dose measurements using Gafchromic films were taken at a depth of 4 mm in SW. Percent depth ionization (PDI) measurements using ion chamber were performed in SW. The profiles generated from film measurements and the PDI plots were compared with those computed using the Acuros algorithm and vendor's data, when available. Preliminary leakage tests were performed using optically stimulated luminescence dosimeters (OSLDs) and the results were compared with Acuros predictions. All applicators were found to be functional with physical dimensions within 1 mm of specifications. For scenario (ii) measurements taken in SW at 5 mm depth and in air at the surface of each applicator were within 10% and 4% agreement with vendor's data, respectively. Compared with Acuros predictions, these measurements were within 6% and 5%, respectively. Measurements taken for scenario (i) showed reduced agreement with both the vendor's data as well as the Acuros calculations, especially when using the 10 mm applicator. The full widths of the measured dose profiles were within 2 mm of those predicted by Acuros at the 90% dose level. The PDI plots and measured leakage dose were in good agreement with vendor's data and Acuros predictions. Based on the dosimetric results, a quality assurance program and procedures for clinical implementation were developed. Treatment planning will be performed using scenario (ii). The 10 mm applicator will not be released for clinical use. A prescription depth of 4 mm is recommended, to ensure full coverage at 3 mm and a minimum dose of 90% of prescribed dose at 4 mm depth.

PACS number(s): 87.55 Qr, 87.56 Da, 87.90 +y

## I. INTRODUCTION

Nonmelanoma skin cancers (NMSCs) are among the most common malignancies, and the United States has one of the highest rates of incidence. An estimated 3.5 million new cases are diagnosed every year in the United States alone and about 580,000 in England, Europe, and Australia.^1^, ^2^ NMSCs occur most commonly in the middle‐aged and older populations. Lately, however, these cancers are increasingly affecting younger populations as well.^(3)^ The American Cancer Society has estimated that melanoma, a more aggressive type of skin cancer, would result in more than 73,000 cases of skin cancer in 2015, accounting for nearly 10,000 deaths of the more than 13,000 skin cancer deaths each year in United States.^(4)^ The incidence of all cancers is projected to increase by an average annual rate of 2.25% through the year 2030, predominantly due to increasing life expectancy, and skin cancer will be no exception.^(5)^ Further improvement in the prevention, diagnosis, and treatment of skin cancer is therefore warranted.

Approximately 80%‐90% of skin cancer cases occur in the head and neck region, 65% involving the face. Treatment in high‐risk anatomical areas involving skin on or near the nose, eyes, ears or mouth can represent a clinical challenge from the perspective of both oncologic and cosmetic outcomes.

Mohs surgery is a proven and well‐known procedure for skin cancer treatment, with five‐year cure rate of 97%‐99%.^(6)^ However, Mohs surgery requires the preparation and examination of frozen pathology, representing a relatively labor‐intensive, time‐consuming, and expensive process. In addition, anatomic and cosmetic concerns can make this option less attractive to patients with lesions in high‐risk anatomic locations, particularly in the setting of equally effective treatment alternatives with less associated cosmetic morbidity.

If detected early, NMSCs can often be treated or controlled effectively with radiation therapy.^(7)^ Of the many forms of treatment for small, shallow skin lesions, a relatively recent approach involves the use of surface applicators in conjunction with a high‐dose‐rate brachytherapy (HDR) source.

Varian has designed two sets of HDR surface applicators. One set with the source horizontally positioned (parallel to the skin surface) contains four applicators with diameters ranging from 30 to 45 mm. The parallel source geometry makes the dosimetry somewhat less challenging. The other set with the source vertically positioned relative to the skin surface contains ten applicators, with dimensions ranging from 10 to 45 mm. Based on our clinical experience, the majority of skin lesions treatable using such applicators are in the range of 15‐25 mm.

The wide range of aperture sizes, and the fact that only the applicators with the source vertically positioned are currently included in the Varian brachytherapy planning system's library of solid applicators, make them appealing to the radiation oncology team. The vertical source orientation results in a challenging dosimetry condition, because of the high gradient in the region of the normal prescription depth (3‐4 mm).

Recently our department purchased the Varian HDR surface applicator set with the source vertically positioned relative to the skin surface. We are not aware of peer‐reviewed published commissioning results or implementation guidelines for this configuration, hence the focus of this paper.

## II. MATERIALS AND METHODS

### A. Commissioning

**Figure 1:**
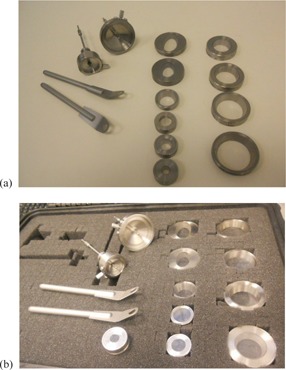
Photo (a) with the components of the Varian surface applicator set with the source vertically positioned and photo (b) of the set placed in a custom‐designed case.

The Varian HDR surface applicators with the source vertically positioned are manufactured with diameters ranging from 10 to 45 mm: eight circular and two ovoid shapes, denoted as follows: SA (small applicators) 10, 15, 20, 25; BA (big applicators) 30, 35, 40, and 45 mm; SAOV (small ovoid‐shaped applicator) with orthogonal short/long diameters of 20/30 mm; and BAOV (big ovoid‐shaped applicator) with orthogonal short/long diameters of 25/45 mm. The set also contains two “shielding for tubus with fixation” components: one for the small circular applicators and one for the large circular plus the two ovoid applicators. The photo in Fig. 1(a) shows the components of the Varian surface applicator set with the source vertically positioned, while the photo in Fig. 1(b) shows the set placed in a custom‐designed case.

The purpose of the commissioning project was to complete a series of measurements for this applicator set in order to validate the vendor data and to develop the procedures for clinical implementation.

#### A.1 Applicator integrity and physical dimensions check

The applicators were visually inspected for signs of damage. The total length of the source guide tube plus the applicator was verified to be 130 cm using the HDR manufacturer's calibrated length wire. Also, an HDR plan with a single dwell position at the distal end was run on the HDR unit (Gamma Med iX, Varian Medical Systems, Palo Alto, CA) using each applicator to ensure that the total length is within the tolerance and there is no obstruction along the path. The physical dimensions and shape for all applicators were checked by direct measurements using a ruler. Also, the shape and wall thickness for the two shielding components and the source channel centering were verified by measurement.

#### A.2 Reference dosimetry using ion chamber measurements

Vendor data (ion chamber and film measurements from 1994) were tabulated at 5 mm depth in water and applicator surface, considering a single dwell position moved back 5 mm, 10 mm, or 15 mm from the center of the first nominal dwell position. A very steep gradient was noticed by the vendor when the source was close to the phantom surface, which may be clinically undesirable.

**Figure 2:**
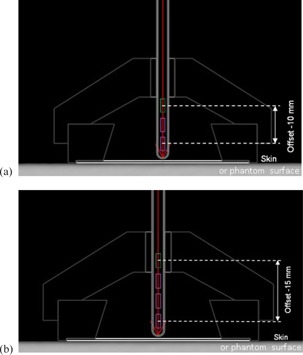
Schematic diagram of the BA30 applicator showing a single dwell position located at (a) −10 mm and (b) −15 mm from the center of the first nominal dwell position.

Consequently, all measurements and calculations described here were performed only for two scenarios: a single dwell position at either ‐10 or −15 mm from the center of the first nominal dwell position (see diagrams in Fig. 2 using the BA30 applicator).

Since there are no published guidelines regarding the output measurements for these applicators, we decided to perform the following tests.

##### A.2.1 Measurements in solid water

Measurements were performed at 5 mm depth in a Solid Water (SW) phantom using a Markus chamber (N23343, PTW Freiburg GmbH, Freiburg, Germany) in a 2.5 cm polystyrene slab with a 5 cm solid water slab for backscatter. The Markus chamber may not be the ideal design for ${\;}^{192}\text{Ir}$ measurements, as it is optimized for beams with energies greater than 2 MeV, but was the most appropriate chamber available to us for these measurements. For the Markus chamber, a calibration factor for ${\;}^{60}\text{Co}$ in water was used (a calibration factor in SW could not be obtained), since a calibration factor for ${\;}^{192}\text{Ir}$ is not available. $P_{\text{pol}}$ and $P_{\text{ion}}$ were measured in SW for all applicators, using a high/low voltage of 300 V and 150 V, respectively, and the “nonpulsed beam” formula for $P_{\text{ion}}$. Corrections were also made to account for the electrometer, temperature, and pressure. The Markus chamber did not have a protection cap during these measurements (only a ring), consequently no correction was needed for the effective point of measurement. Any potential volume averaging effects due to the chamber dimensions were not considered in this study.

The reading in SW was used to obtain the dose to water as follows:

(1) $$Dose\;to\;water=M_{raw}\;in\;SW\times N_{D,w}^{Co_{60}}\times P_{pol,SW}\times P_{ion,SW}\times P_{el}\times P_{T,P,SW}\times phantom\;factor.$$

**Figure 3:**
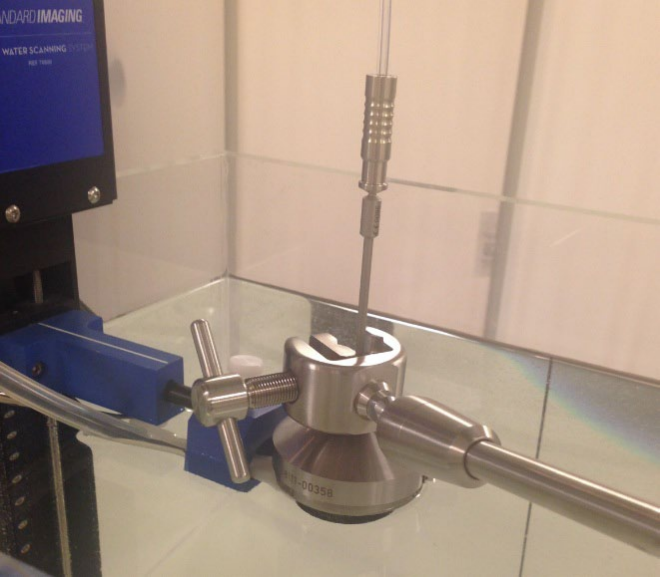
Experimental setup for dose measurements in water used to obtain the SW‐ to‐ water phantom factor.

A solid water‐to‐water phantom factor was previously obtained for a similar setup.^(8)^ The phantom factor was checked for one large (BA45) and one small (SA25) applicator using the methodology described in the literature.^(8)^ A 1D motorized water tank was used for these measurements. The applicator was centered at the water surface over the Markus chamber, which was moved down from the water surface such that the effective point of measurement was located at 5 mm depth. A 1 mm protective cap was used for the chamber and its thickness was included in the depth assuming it is water equivalent (see Fig. 3 for the setup details). For both applicators, the dose in water measured as described above was compared with the vendor's data and the dose predicted by the Acuros BV Grid‐Based Boltzmann Solver (GBBS) code used in the BrachyVision 11 (Varian Medical Systems) treatment planning system for heterogeneity corrected dose computation.

The phantom factor was obtained using the following equation:

(2) $$phantom\;factor=\frac{Dose\;in\;water}{Dose\;in\;SW}=\frac{M_{raw}\;in\;W\times N_{D,w}^{Co_{60}}\times P_{pol,w}\times P_{ion,w}\times P_{el}\times P_{T,P,w}}{M_{raw}\;in\;SW\times N_{D,w}^{Co_{60}}\times P_{pol,sw}\times P_{ion,sw}\times P_{el}\times P_{T,P,sw}}$$

A phantom factor of 1.07 was calculated and used in Eq. (1) above for all applicators, to convert the corrected reading in SW to dose to water.

**Figure 4:**
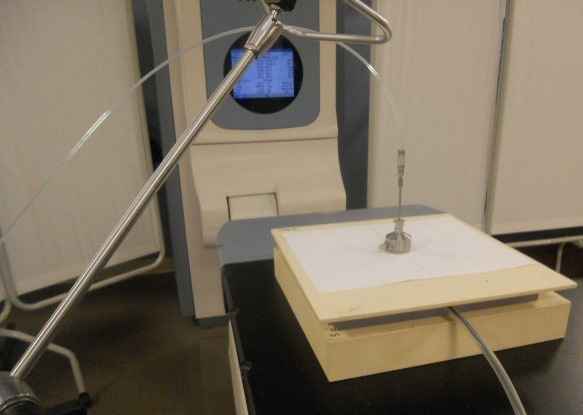
Experimental setup for the measurements at 5 mm depth in a Solid Water phantom.

Measurements in SW were performed on the couch of a Conventional Simulator, which resides in the same room with the HDR unit. The lasers and graticule were used to center the chamber. For these measurements, paper templates were centered on the top of the phantom over the ion chamber using the lasers and graticule to achieve alignment, and then the applicator was placed over the template and connected to the HDR unit (see Fig. 4 for the setup details). The results were compared with vendor data and calculated values using the Acuros algorithm.

##### A.2.2 Measurements in air

**Figure 5:**
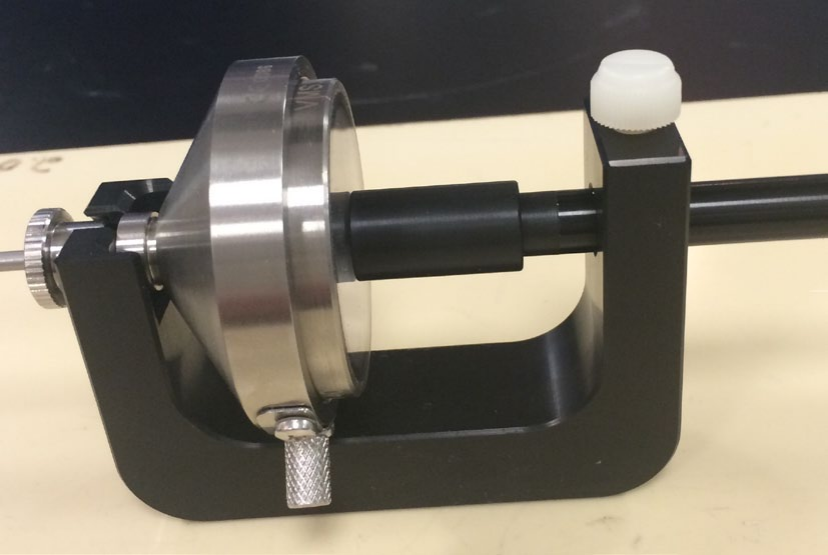
Experimental setup for the measurements in air at the surface of the applicators.

Dose measurements at the applicator surface were performed in air using an Exradin A20 ion chamber and a Standard Imaging alignment jig (Standard Imaging Inc., Middleton, WI) designed for such measurements (see Fig. 5 for the setup details). The calibration factor ($N_{k}$) for ${\;}^{192}\text{Ir}$ was obtained through interpolation between the factors for ${\;}^{137}\text{Cs}$ and a 250 kV beam. For this chamber, $P_{\text{pol}}$ and $P_{\text{ion}}$ were measured in air for all applicators considering a high voltage of 300 V, a low voltage of 150 V, and using the “nonpulsed beam” formula for the $P_{\text{ion}}$. Corrections were also made to account for the electrometer, temperature, and pressure. The corrected reading was converted to dose (as if the applicator were in contact with a water phantom) following the methodology described in the AAPM Task Group 61 report:^(9)^

(3) $$D_{w,z=0}=M_{raw}\times P_{pol}\times P_{ion}\times P_{el}\times P_{T,P}\times N_{k}\times B_{w}\times P_{stem,air}\times {\left[{\left(\frac{\mu_{en}}{\rho }\right)}_{air}^{w}\right]}_{free\;air}\times POM,$$

where

(4) $$POM(po\text{int}\;of\;measurement)={\left(\frac{SSD+d}{SSD}\right)}^{2}.$$

Here *SSD* is 14.7 mm and 19.7 mm for a single source located at −10 mm and −15 mm from the center of the first nominal dwell position, respectively, and *d* is 3.4 mm [1.6 mm ion chamber cap thickness^(10)^ and 1.8 mm shift for the point of measurement relative to the ion chamber tip^(11)^]. The plastic inset thickness is 1.7 mm (included in the SSD).

In Eq. (3) above, the backscatter factor, $B_{w}$, was assumed to be 1.08. This value was based on the data published in the AAPM Task Group 61 report^(9)^ for an energy of ∼300 kV, with a similar field size range and SSD as used in our experiment. While the TG‐61 report was designed for X‐ray spectra with 40‐300 kVp, we believe it is the most appropriate formalism for the work described in this section.

The stem correction factor $P_{\text{stem},\text{air}}$ was previously measured for the A20 ion chamber for a field size range of 10‐50 mm, comparable to those used for these measurements.^(11)^ A value of 1.02 (almost independent of the field size) was obtained for this factor^(11)^ and used in Eq. (3) above.

For the mass energy absorption coefficient ${\left[{\left(\frac{{\bar{\mu }}_{en}}{\rho }\right)}_{air}^{w}\right]}_{free\;air}$ a value of 1.10 published for the ${\;}^{192}\text{Ir}$ source^(12)^ was used in Eq. (3) for surface dose calculation. The Exradin A20 ion chamber was designed by Standard Imaging for measurements with the HDR ${\;}^{192}\text{Ir}$ surface applicators and recommended by Varian. Similar setup for surface applicators dose measurements was previously used by other groups^10^, ^11^ for either source vertically or horizontally positioned relative to the skin surface. Future work will be performed using a very thin‐walled, well‐guarded parallel plate detector.

Given the fact that, for some of the parameters in Eq. (3) above there is no published value for the HDR ${\;}^{192}\text{Ir}$ source, therefore requiring some approximations, the in‐air surface dose measurements were not intended to be “absolute dosimetry” tests. They are very convenient to perform and can be used as a constancy check for the annual quality assurance testing.

The results obtained with this setup were compared with vendor data and calculated values using Acuros.

#### A.3 Acuros calculations

The applicators are made from tungsten (a high‐Z material), so they cannot be CT‐scanned for treatment planning. Our physics group decided to perform the treatment planning using the Varian library for solid applicators and the Acuros BV GBBS algorithm for heterogeneity corrected dose calculations.

A SW phantom was scanned and the Acuros BV algorithm was used to compute the dose at 5 mm depth and at surface for all surface applicators, using a resolution of 0.05 cm and assuming medium is water. The dwell times were selected from the vendor data such that the predicted dose at 5 mm depth would be 3.0 Gy. Resulting dose values were compared with the vendor measurements and also with our measurements described above.

#### A.4 Relative dosimetry using Gafchromic film measurements

Film measurements were performed for all applicators at 4 mm (prescription) depth in a SW phantom, considering a single dwell position located at −15 mm from the center of the first nominal dwell position. Measurements were performed on the conventional simulator couch. The lasers and graticule were used to center the applicator over the film. The dwell time was set such that the dose at depth would be approximately 1.0 Gy for the large applicators and 2.0 Gy for the small applicators. After a minimum time interval of 24 hours from exposure, the film was scanned in transmission mode with a resolution of 300 dpi using an Epson Perfection V700 Photo dual lens system (Epson America, Long Beach, CA) and analyzed with the ImageJ software (ImageJ, National Institutes of Health, Bethesda, MD) to obtain the profiles. Profiles were taken along various directions on the film in order to verify the dosimetric source centering. Their shape, width, and penumbra dose falloff were compared. Further manipulation (i.e., normalization) was performed using the Origin software (OriginLab Corporation, Northampton, MA).

Theoretical profiles at the same depth were also obtained for each applicator using the Acuros software (calculations were performed in water with a resolution of 0.05 cm).

The width of the 90% dose level was obtained for all applicators from both the measured and Acuros‐based profiles.

#### A.5 Relative dosimetry using ion chamber measurements

Percent depth ionization (PDI) measurements were performed in a SW phantom for one big (BA45) and one small applicator (SA25), using the same Markus chamber used for reference dosimetry, embedded in a 2.5 cm polystyrene slab with a 5 cm SW slab for backscatter.

Measurements were performed on the couch of a conventional simulator, using the lasers and graticule to center the chamber and the applicator, as described in the Materials & Methods section 2.1) above.

We did not attempt to obtain the factors to convert the ionization to dose, since they may be depth dependent. The normalized measured values (i.e., the PDI) were compared with vendor's percent depth dose (PDD) data and Acuros PDD predictions.

#### A.6 Leakage check using optically stimulated luminescence dosimeters (OSLDs)

OSLDs were used to experimentally verify the leakage for the Varian surface applicators. Unscreened OSLDs were screened in‐house, using a 1.00 Gy exposure to 6 MV X‐rays to determine sensitivity adjustment factors for each dosimeter and then the OSLD reader system was calibrated using 6 MV X‐rays. Previous *in vivo* measurements of peripheral surface dose performed at our institution for HDR ${\;}^{192}\text{Ir}$ skin flap treatments indicated absolute dose agreement within ∼10% of calculated dose at distances greater than 1 cm.

**Figure 6:**
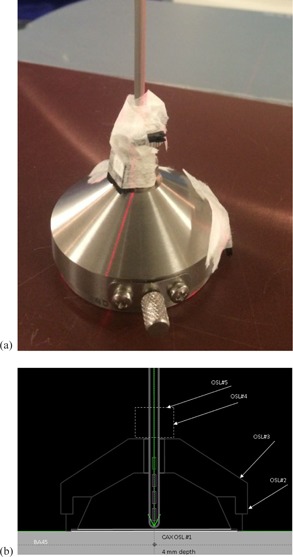
Experimental set‐up for (a) leakage measurements using OSLDs and (b) schematic diagram with the setup and OSLDs labeling.

Leakage tests were performed for three applicators: BA45, SA25, and SA15. The measurements were performed on a conventional simulator couch, using the lasers and graticule for alignment. A 5 cm SW slab was used for backscatter, and then a 0.5 cm bolus slab was placed over the SW slab. An OSLD was positioned over the bolus slab and centered using the lasers and graticule. A 4 mm SW slab (prescription depth) was placed over the OSLD and the applicator was centered over the SW slab. Four OSLDs were taped on the applicator surface (see Fig. 6(a) for the setup and the diagram in Fig. 6(b) for labeling) and an HDR plan was run considering a single source position located at −15 mm from the center of the first nominal dwell position. The total dwell time was set to 200 s.

The OSLD readout was performed a few minutes after exposure. The data (relative to the CAX dose measured with the OSLD placed at 4 mm depth) was compared with the leakage estimated with the Acuros algorithm for the same scenario, using a resolution of 0.05 cm.

## III. RESULTS & DISCUSSION

### A. Applicator integrity and physical dimensions check

All applicators were in good condition and their dimensions were within 1 mm from nominal values. The shape was correct (circular, ovoid) for all applicators and shielding components and the source channel was found to be physically centered.

### B. Reference dosimetry using ion chamber measurements

The results for the output measurements and calculations are summarized below.

#### B.1 Measurements at 5 mm depth in SW

The measurements performed at 5 mm depth in water in order to validate the previously measured SW‐to‐water phantom factor were in good agreement with vendor data (within 5%‐10% for small/large applicators) and Acuros predictions (within 3%). For a source located at −10 mm from the center of the first nominal dwell position, the agreement with vendor data was within ∼15%, while with Acuros calculations it was within ∼5%. For a source located at −15 mm, the agreement with vendor data was within 10%, except for the 10 mm applicator (15%), while with Acuros calculations it was within ∼4%, except for the 10 mm applicator (7%). Table 1 shows the percent difference of the dose measured at 5 mm depth in SW and manufacturer data/Acuros calculations, for the two scenarios described here.

**Table 1:** Percent difference between the dose measured at 5 mm depth in solid water and manufacturer data/Acuros calculations for a single dwell position located at −10 mm or −15 mm from the center of the first nominal dwell position

*Applicator*	*% diff. manuf*. −10 mm	*% diff. Acuros* −10 mm	*% diff. manuf*. −15 mm	*% diff. Acuros* −15 mm
SA10	8.6	0.7	14.4	−7.0
SA15	−7.4	−0.8	−7.7	−4.1
SA20	−9.3	−1.0	−7.0	−1.4
SA25	−11.3	−4.2	−5.8	0.9
BA30	−13.9	3.3	−10.0	−3.9
BA35	−11.7	1.8	−7.5	−2.0
BA40	−12.0	3.3	−6.7	−0.7
BA45	−12.2	0.7	−9.7	−1.1
SAOV	−10.8	5.1	−5.0	2.0
BAOV	−11.2	2.4	−9.4	1.3

#### B.2 Surface dose measurements in air

For a source located at −10 mm the agreement with vendor data was within 8% (except for the 10 mm applicator), while with Acuros calculations it was within ∼16%. For a source located at −15 mm, the agreement with vendor data was within 6%, except for the 10 mm applicator, while with Acuros calculations it was within 5%. See Table 2 for the percent difference of the surface dose measured in air and manufacturer data/Acuros calculations, for the two scenarios mentioned above regarding the source dwell position.

**Table 2:** Percent difference between the surface dose measured in air and manufacturer data/Acuros calculations for a single dwell position located at −10 mm or −15 mm from the center of the first nominal dwell position

*Applicator*	*% diff. manuf*. −10 mm	*% diff. Acuros* −10 mm	*% diff. manuf*. −15 mm	*% diff. Acuros* −15 mm
SA10	26.8	10.5	29.3	0.0
SA15	7.7	15.4	2.2	1.6
SA20	5.0	11.0	−2.1	−0.5
SA25	4.6	10.2	1.9	4.7
BA30	−0.6	13.8	−2.8	2.0
BA35	−1.2	12.0	−3.7	−0.7
BA40	−4.2	9.2	−5.9	−3.0
BA45	−4.9	7.4	−5.5	−0.1
SAOV	2.7	16.3	−2.9	0.3
BAOV	−0.6	10.7	−3.6	1.4

### C. Acuros calculations

The Acuros license was installed and a functionality test was performed, followed by a simple acceptance test designed to compare the Acuros dose values against vendor data. The agreement was within the ±2% tolerance. More thorough tests were designed for commissioning.^(13)^ The results have demonstrated acceptable performance of the Acuros BV GBBS algorithm and, based on these findings, the software was released for clinical use for the Varian surface applicators dose calculation.

For a source located at −10 mm, the agreement with vendor data at surface was within 15%. For a source located at −15 mm, the agreement with vendor data at surface was within 5.5%, except for the 10 mm applicator. See Table 3 for the percent difference of the surface dose calculated with Acuros and manufacturer data, for the two scenarios regarding the source dwell position.

**Table 3:** Percent difference between the surface dose calculated with Acuros and manufacturer data for a single dwell position located at −10 mm or −15 mm from the center of the first nominal dwell position

*Applicator*	*SA10*	*SA15*	*SA20*	*SA25*	*BA30*	*BA35*	*BA40*	*BA45*	*SAOV*	*BAOV*
%diff. mnf −10 mm	14.7	−6.7	−5.4	−5.1	−12.6	−11.8	−12.2	−11.4	−11.7	−10.2
%diff. mnf −15 mm	29.3	0.5	−1.6	−2.7	−4.7	−3.0	−3.0	−5.4	−3.2	−5.0

### D. Relative dosimetry using Gafchromic film measurements

The profiles obtained in various directions on the film looked very similar, with comparable width and symmetric dose falloff on both sides, confirming a dosimetric centering of the HDR source.

**Figure 7:**
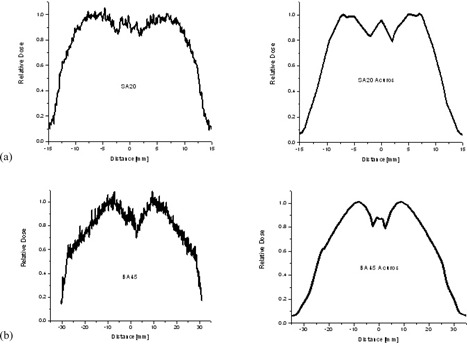
Plots with the measured and Acuros calculation‐based profiles for the (a) SA20 and (b) BA45 applicators.

As an example, the measured and Acuros calculation‐based plots with the profiles for the SA20 and BA45 applicators are shown in Fig. 7(a) and (b). Table 4 shows the full width at 90% dose level for the profiles at 4 mm depth, obtained from Gafchromic film measurements and Acuros calculations. The width of the 90% dose level from both the measured and Acuros‐based profiles agreed within ∼2 mm for all applicators. The steep gradient makes the measured profiles noisy. Based on our experimental observations, a 1.0 mm depth setup error would result in approximately 10% variation in dose. Of note, a 0.5 mm spatial shift could result in as much as a 5% change in the Acuros‐calculated dose. The highest spatial resolution available for the Acuros dose calculation grid is 0.5 mm.

**Table 4:** Full width (in mm) at 90% dose level based on Gafchromic film measurements and Acuros calculations

								*BAOV*	*BAOV*	*SAOV*	*SAOV*
*Applicator*	*SA15*	*SA20*	*SA25*	*BA30*	*BA35*	*BA40*	*BA45*	*long*	*short*	*long*	*short*
Meas. Width (mm)	18.2	18.8	19.6	30.0	30.2	30.5	31.2	28.8	28.9	30.3	24.3
Acuros‐calculated Width (mm)	16.7	17.0	17.6	28.6	28.5	28.9	28.9	28.0	27.0	28.7	23.4

### E. Relative dosimetry using ion chamber measurements

**Figure 8:**
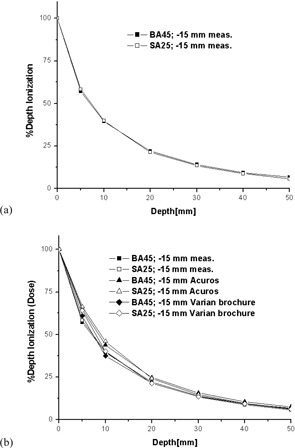
PDI/PDD plots for the BA45 and SA25 applicators: (a) measured PDI data and (b) measured PDI data plotted against PDD vendor's data and Acuros predictions.

Figure 8(a) shows the PDI plots for the two applicators (BA45 and SA25) used for measurements, considering a single dwell position located at −15 mm from the center of the first nominal dwell position. Both plots were normalized to 100% at the surface. The two curves are almost identical, in agreement with the trend of the vendor's data which show almost identical PDD plots for all applicators when using the same source position. Figure 8(b) shows the two measured PDI plots described above and the corresponding PDD plots obtained from vendor's data and computed using Acuros (0.05 cm resolution). The measured PDI results are in good agreement with both the vendor and the Acuros data, especially in the range of prescription depth (3‐4 mm). Acuros calculation was also performed for a single‐source position located in air at −15 mm from the center of the first nominal dwell position and considering a 2 mm offset away from the water phantom surface to mimic the thickness of the plastic insets of the surface applicators. The applicators do not seem to significantly alter the spectrum of the HDR source because the computed PDD was almost identical with that obtained for the surface applicators with the same source configuration (only slightly larger values at greater depths).

**Figure 9:**
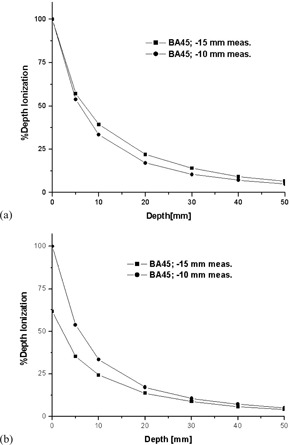
PDI plots for the BA45 applicator for two scenarios (i.e, a single source position at either ‐10 or −15 mm from the center of the first nominal dwell position): (a) normalized to the surface and (b) normalized to the surface value when the source was located at −10 mm.

For the BA45 applicator, measurements were also performed for a single‐source position located at −10 mm from the center of the first nominal dwell position. Figure 9(a) shows the BA45 PDI plots normalized to 100% at the surface when the source is located at −15 mm and −10 mm. Figure 9(b) shows the same plots, but the data for the source located at −15 mm were normalized against the data for the source located at −10 mm, for which the surface PDI value was set to 100%. The dose falloff is more pronounced for the source located at −10 mm, in agreement with vendor's data trend.

### F. Leakage check using OSLDs

Our preliminary leakage measurements showed that the leakage is dependent on the applicator holder used (large versus small) and is higher for the smaller applicators. In position 2 (see diagram from Fig. 6(b)) the leakage is in the range 3%‐6% of prescription dose for all three applicators used, in good agreement with the 4% value predicted by Acuros. For the OSLD placed in position 3 (applicator holder oblique surface), the leakage is ∼2% for the large applicators and 28%‐40% for the small applicators, in agreement with Acuros predictions.

The OSLDs placed close to the source channel in positions 4 and 5 showed high leakage values (more than 100% prescription dose for position 4 and 35%‐60% prescription dose for position 5), in agreement with the trend of Acuros data.

## IV. CLINICAL IMPLEMENTATION AND QUALITY ASSURANCE (QA) PROGRAM A. Clinical implementation

Consistent with our department's approach for any new technology, a working group was established to develop a clinical implementation methodology for the Varian surface applicators. The working group, composed of two physicists, a simulation therapist, a radiation oncologist and a nurse, developed the workflow guidelines and step‐by‐step procedures and checklists for the entire clinical process, from simulation to treatment. When the entire commissioning work was finalized and the clinical process validated, an in‐service was performed in the department prior to our first case to ensure that all staff members were familiar with the clinical process, equipment, workflow, checklists, and documentation.

We are currently awaiting the first patient treatment. The clinical implementation procedures described below were generated in advance to be ready for the coming cases.

### A.1 Simulation

The simulation procedure describes in detail the patient positioning approach, immobilization devices to be fabricated (such as thermoplastic mask, vacuum‐bag molds), target marking, applicator selection, and proper placement of the universal clamping device used to keep the heavy applicator rigidly in place during treatment. The procedure document also establishes the technical parameters to be used for scanning, including slice thickness (1 mm) and field of view (FOV), which should be limited to the target and adjacent critical structures in order to optimize spatial resolution. The surface applicators are made of tungsten, so CT scanning cannot be performed with the applicators in place. Consequently, the FOV should also include some air where the applicator will be placed for treatment. Any need for shielding (e.g., eye, nose) should also be determined during simulation. Setup reproducibility is a priority, given the weight of the applicator.

The surface applicators cannot be sterilized. Tegaderm (3M Health Care, St Paul, MN) or thin plastic wrap (if patient is allergic to Tegaderm) will be used as a barrier between the applicator and the skin. No air gap should be left between the plastic barrier and the skin. The radiation oncologist will mark the target on the patient skin and will check whether the target surface is sufficiently flat for good contact of the surface applicator and skin. To ease the planning process and treatment setup, four radiopaque markers (”BBs”) will be placed right outside the delineated target to mark two orthogonal diameters for the round applicators. For the ovoid‐shaped applicators, a CT wire will be placed on the skin around the delineated target, along with four BBs used to define two orthogonal (long and short) diameters.

As seen in Table 4, the width of the 90% dose level at the prescription depth of 4 mm is not equal to the nominal dimension of a given applicator. Plots with the measured and Acuros‐calculated profiles for all applicators are printed and readily available in the simulation room to facilitate the applicator selection process. Also, plastic templates were generated for all applicators (round and ovoids), with the diameters given by the width of the 90% dose level at 4 mm depth. To ensure the target is covered at the prescription depth, a plastic template with an appropriate size will be selected and placed over the delineated target. The applicator will also be placed over the delineated target to check the skin contact, but keeping in mind that the area covered by the 90% dose level is not given by the applicator's nominal dimension.

### A.2 Planning

A CT‐based plan will be generated using the BrachyVision 11 treatment planning system equipped with the Acuros BV algorithm, with a resolution of 0.05 cm and the appropriate CT density for the medium and applicator, which is loaded from the Varian library of solid applicators. The BBs and the wire placed over the skin during simulation help with the correct centering of the surface applicator as prescribed by the radiation oncologist. (Prior to dose calculation, the density of BBs [and wire when applicable] are set to zero.) The planning target volume (PTV), defined as the delineated target area to a depth of 4 mm, and skin will be contoured and a plan generated using a single dwell position located at −15 mm from the center of the first nominal dwell position. The dose will be prescribed at 4 mm depth. Other organs at risks (such as lens of the eye) will be contoured as appropriate. If the PTV is not properly covered, the physicist will check if the coverage is improved by using a different applicator and discuss the results with the radiation oncologist. The dose‐volume histograms (DVH) for the PTV and skin will be analyzed and the PTV D90%, V90%Rx, and V100%Rx and skin D1cc and V145%Rx recorded.

The following DVH criteria were established: PTV D90%>100%Rx,PTV V90%Rx=100%, skin D1cc<125%Rx. The maximum dose (D0.1cc) for the other defined critical structures will also be recorded. The physicist and radiation oncologist will jointly determine if the maximum dose is acceptable.

### A.3 Treatment

**Figure 10:**
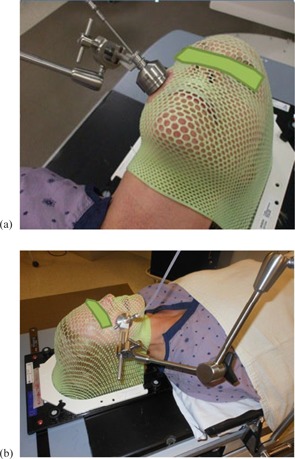
Photo (a) with the setup showing the universal clamping device arm holding the surface applicator rigidly in place during treatment and photo (b) showing the treatment setup using a universal clamping device mounted on the treatment couch side bar.

The treatment procedure explains in detail the treatment process, including immobilization devices to be used, reproducibility of the setup, plastic barrier placement, applicator verification (size and orientation) and placement, and proper connection to the HDR unit via a source guide tube (SGT). The treatment plan generated for the first fraction will be used for all subsequent treatments, so the setup reproducibility is essential. For a tumor located on the face, the mask will be cut during simulation to uncover the PTV and surrounding skin, ensuring that the applicator can be placed in close contact with the skin during treatments. Figure 10(a) shows the universal clamping device arm holding the surface applicator rigidly in place during treatment and Fig. 10(b) shows the treatment set‐up and the universal clamping device mounted on the treatment couch side bar.

Treatment checklists were generated for physicists/radiation oncologists and radiation therapists to ensure all steps are properly followed.

### B. Quality assurance program

Based on the findings described here, the following quality assurance (QA) program was implemented for the brachytherapy procedures using the Varian surface applicators:

a) AQA plan is generated for an independent check considering the medium as water and keeping the same total dwell time as for the patient plan. The computation is performed using Acuros, with a resolution of 0.05 cm. A total dwell time based on vendor data in water, using an Excel (Microsoft, Redmond, WA) spreadsheet developed for this purpose, is obtained for the same dose as in the QA plan, at the prescription depth of 4 mm. The agreement between the two total dwell time values should be within 10%.

b) SGTs are included in the normal SGT QA rotation schedule.

c) Annually, as a constancy check, the in‐air “surface dose” output of each applicator (except the 10 mm applicator) will be measured using the A20 chamber in its special measurement jig considering a single dwell position located at −15 mm from the center of the first nominal dwell position. Agreement within 10% of the Acuros‐calculated value will be considered acceptable.

## V. CONCLUSIONS

Our work has demonstrated that the vertically oriented Varian surface applicators have predictable dosimetric characteristics accurately modeled by the Acuros BV algorithm, and are, therefore, appropriate for clinical use for small, shallow skin lesions. The reduced agreement between our measurements and vendor/Acuros data for a source located at −10 mm from the center of the first dwell position (especially when using the SA10 applicator) is caused by the very steep dose gradient's significant impact on even small setup uncertainties.

Based on the aforementioned tests and dosimetric results, we have the following recommendations:

1. The 10 mm applicator will not be released for clinical use.
2. Treatment planning will be performed using a single dwell position located at −15 mm from the center of the first nominal dwell position.
3. The dose will be prescribed at 4 mm depth to ensure full coverage at 3 mm and a minimum dose of ≥90% of prescribed dose at 4 mm depth.
4. As a conservative approach, the applicator selection will be based on the 90% dose level width at 4 mm depth from Acuros plans.
5. Careful consideration of patient positioning (including possibly lateral decubitus) at the time of CT simulation is important to ensure reproducible setup of the surface applicator.
6. Given our preliminary results for leakage dose, careful consideration of applicator positioning relative to normal tissue, critical structures, and skin folds is important. Any need for shielding and/or OSLD measurements will be assessed during CT simulation.
7. High‐resolution CT data (limited axial FOV and 1 mm slice thickness) are important to ensure accurate dose calculation given the 0.05 cm dose voxel dimension.

## ACKNOWLEDGMENTS

We would like to thank our colleague Deborah Savini for her help with the procedures for the clinical implementation of this treatment modality in our clinic, and our colleague Mike Talmadge for his help with leakage measurements.

## COPYRIGHT

This work is licensed under a Creative Commons Attribution 4.0 International License.
